# Larval behaviour, dispersal and population connectivity in the deep sea

**DOI:** 10.1038/s41598-020-67503-7

**Published:** 2020-06-30

**Authors:** Stefan F. Gary, Alan D. Fox, Arne Biastoch, J. Murray Roberts, Stuart A. Cunningham

**Affiliations:** 1 SAMS, Scottish Marine Institute, Oban, Argyll PA37 1QA UK; 2 School of GeoSciences, The Grant Institute, University of Edinburgh, James Hutton Road, The King’s Buildings, Edinburgh, EH9 3FE UK; 3 GEOMAR Helmholtz Centre for Ocean Research Kiel, Düsternbrooker Weg 20, 24105 Kiel, Germany; 4 Kiel University, Christian-Albrechts-Platz 4, 24118 Kiel, Germany; 5 Parallel Works Inc., 222 Merchandise Mart Plz. Suite 1212, Chicago, IL 60654 USA

**Keywords:** Biooceanography, Ecological modelling, Ecological networks, Marine biology, Physical oceanography

## Abstract

Ecosystem connectivity is an essential consideration for marine spatial planning of competing interests in the deep sea. Immobile, adult communities are connected through freely floating larvae, depending on new recruits for their health and to adapt to external pressures. We hypothesize that the vertical swimming ability of deep-sea larvae, before they permanently settle at the bottom, is one way larvae can control dispersal. We test this hypothesis with more than $3\times 10^{8}$ simulated particles with a range of active swimming behaviours embedded within the currents of a high-resolution ocean model. Despite much stronger horizontal ocean currents, vertical swimming of simulated larvae can have an order of magnitude impact on dispersal. These strong relationships between larval dispersal, pathways, and active swimming demonstrate that lack of data on larval behaviour traits is a serious impediment to modelling deep-sea ecosystem connectivity; this uncertainty greatly limits our ability to develop ecologically coherent marine protected area networks.

## Introduction

Many ocean bottom dwelling species release their larvae into the water column to be recruited into the local population and spread further afield to support remote populations and colonize new sites. During transit, larvae may exhibit a range of behaviours to maximize their immediate survival (finding food and avoiding predation) and their long-term survival (finding a spot to settle). An understanding of larval pathways and downstream colonization informs studies of natural and man-made networks, feeds into the design of Marine Protected Area networks, and ultimately impacts how the marine environment is used^1^.

The connectivity of marine ecosystems is fundamental to survival, growth, spread, recovery from damage, and adaptation to changing conditions on ecological and evolutionary timescales^2–4^. The benefits of connectivity information for conservation management is supported by empirical evidence^5,6^. Marine connectivity knowledge is rapidly expanding, with recent seascape genetics approaches that combine particle tracking with genetic techniques^7–10^.

Direct evidence of deep sea population connectivity, through tagging and tracking, is exceedingly rare; indirect estimates of connectivity by genetic methods, elemental fingerprinting, or particle modelling are essential tools. Particle modelling simulates the tracks of larvae within a hydrodynamic model. In the deep sea, this approach is limited to relatively few studies^11–16^.

Modelled connectivity and dispersal estimates depend on larval life history traits. For fish species, pelagic larval duration (PLD) shows a direct correlation with habitat depth^17^. However, fewer than 100 species living deeper than 200 m have published PLD estimates; over 80% of these are echinoderms on sedimentary slopes^18^. In addition to PLD, dispersal depth is also a critical parameter^15,19^. However, for both parameters there are large uncertainties in both their values and interactions, and there are even potentially more parameters to study including mortality, predation, temperature effects and energy limitation.

**Figure 1:**
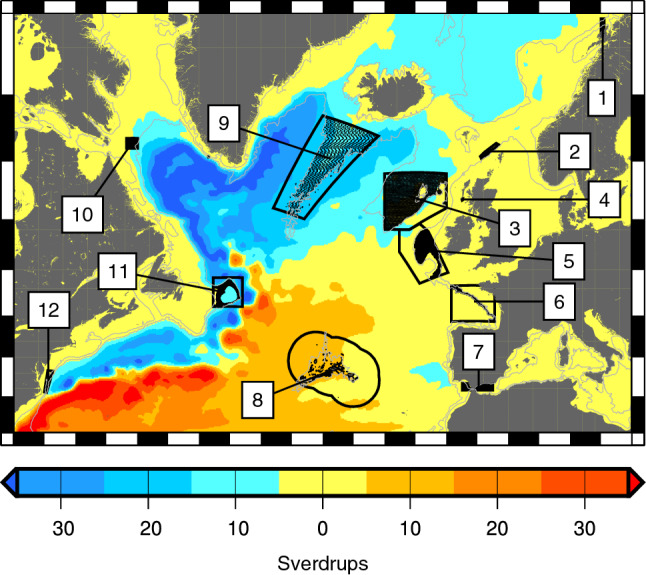
The modelled particle release sites (black points) are within study areas (1–12, black outlines) superimposed on the mean barotropic streamfunction calculated from 50 years (1959–2008) of VIKING20 hindcast. Depth-average currents are directed along lines of constant streamfunction (streamlines) with higher streamfunction values to the right when facing downstream. Current strengths ($1\,\hbox {Sverdrup} = 1\times 10^{6}\,\hbox {m}^{3}\hbox {s}^{-1}$) are inversely proportional to the spacing between streamfunction contours. The case study areas are 1: LoVe Observatory (LoVe), 2: Faroe-Shetland Channel (FSC), 3: Rockall Bank (RB), 4: East Mingulay (EM), 5: Porcupine Seabight (PS), 6: Bay of Biscay (BoB), 7:Gibraltar (Gib), 8: Azores (Az), 9: Reykjanes Ridge (RR), 10: Davis Strait (DS), 11: Flemish Cap (FC), and 12: US Canyons (USC). See also Table 1.

We hypothesize that the vertical swimming ability of deep-sea larvae, before they permanently settle at the bottom, is an important way larvae can control dispersal. To test this hypothesis, we perform basin-scale particle tracking from 12 zones across the North Atlantic Ocean (Fig. 1) selected to incorporate the diversity of sensitive Atlantic deep-water ecosystems. We systematically quantify which larval behaviours, and combinations of behaviours, have the strongest influence on modelled dispersal in the deep sea. We also explore whether these behaviours can change larval pathways, ultimately impacting where larvae settle. This systematic approach extends previous work by accounting for the large uncertainties in many larval life history traits while simultaneously defining the envelope of larval behaviour impacts.

## Methods

Particles are simulated with the Ariane software (available from http://stockage.univ-brest.fr/~grima/Ariane/doc.html,^20,21^) modified to include independent, age-related, vertical motion of particles^22^. The modified Ariane is driven by the velocity, potential temperature, *T*, and salinity, *S*, fields output from the VIKING20 configuration^23^ of the NEMO ocean model^24^.

### VIKING20 hydrodynamic model: description and verification

Used here as input for the Lagrangian dispersal study was VIKING20, an eddy-rich ($0.05^{\circ }$ or 2 km to 4.5 km in the region of interest) ocean/sea-ice model performed under past atmospheric forcing using the CORE2 data set^25^ of the years 1958-2009. VIKING20 is configured on the ORCA family of tripolar grids avoiding a singularity at the North Pole^26^, with the $0.05^{\circ }$ resolution grid covering the North Atlantic two-way nested^27^ within a $0.25^{\circ }$ resolution global ocean. Model vertical discretisation is on 46 z-levels (fixed in depth) with partial cells at the bed for improved representation of bathymetry. Level thicknesses range from 6 m at the surface to 250 m at 5000 m depth (about 30 m at 250 m and 200 m at 2000 m depth). VIKING20 has been demonstrated to realistically simulate the the subpolar North Atlantic^23^ including details of the challenging regional and western boundary current systems^28–30^.

Like many modern ocean models, VIKING20 is optimized for the upper water column. Near-bed flows, crucial to modelling the settling phase of larval life, are typically affected by larger vertical resolution at depth, not resolving the bottom boundary layer. Also not included is the simulation of tides which would lead to locally enhanced mixing and small-scale turbulence^31, 32^. Nevertheless, studies simulating the dispersal of deep-sea mussels^16^ or methanotrophic bacteria^33^ have demonstrated the success of VIKING20 when compared to genetic and in situ data. Despite a general impact of temporal resolution^34^, variable bottom flows are well represented by five-day average fields of the three-dimensional velocities. The mean horizontal velocities are direct output from the model and corresponding vertical velocities are calculated using the model 3D continuity equation.

A direct comparison of individual modelled Lagrangian particle tracks with real-world drifter tracks is impossible - uncertainties in the initialization of the model and missing model dynamics mean that the simulated and real flow fields are never the same and Lagrangian particles in each will quickly diverge. However, when large numbers of virtual particles are released, as we do here, ensembles of particle tracks have proved adept at reproducing distributions of drifting buoys, marine litter, and nutrient concentrations^35–39^. The current generation of global- and basin-scale models at least partially resolve mesoscale eddies, and shows some success in reproducing large-scale and regional circulation patterns, and are therefore able to reproduce coherent structures, patch stretching and straining, attractors and barriers^40^ which impact particle dispersal.

### Particle track calculations

The Ariane particle tracking code finds the analytical solution for particle pathways through model gridcells; we store the updated particle positions at 5-day intervals. Modifications have been added to Ariane to include independent, age-related, vertical motion of each tracked particle, simulating the ability of organisms to actively swim vertically or control their buoyancy. This is done by adding the ‘swimming’ velocity to the vertical velocity calculated from the model for each particle at each timestep. Near the bed, downward swimming is phased out smoothly in the next-to-bottom gridbox and set to zero in the bottom gridbox, upward swimming is phased out smoothly at the target depth and zero above this depth. Near-bed particles are still advected by the model flows, particles advected back into the model interior will resume downward swimming.

Virtual particles were spawned from the 12 ATLAS project case study sites (Fig. 1). For the ATLAS project, these case study sites were selected on the basis of: proximity to blue growth activities, the presence of ATLAS focal ecosystems (cold water corals, sponges and hydrothermal vents), and data availability (see Table 1 for details). These sites were considered suitable for the current modelling study since the site distribution incorporates the diversity of sensitive Atlantic deep-water ecosystems and follows the major Atlantic current patterns. This enables us to test our hypothesis, and study the effects of larval behaviour, in a range of dynamical regimes representative of the 200 m to 2000 m depth range, including offshore banks and seamounts, east and west Atlantic shelf slope, the mid-Atlantic ridge and shallower sites on the shelf.

At each release time, two particles were released at every VIKING20 grid node that lies within the Case Study polygon and the isobath range (Table 1), one particle from near the bottom and one at the top of each bottom grid box (between a few metres and 200 m above the bed, the different release depths had no impact on the dispersal statistics). Particles were released at 200 discrete times: one burst of particles in the middle of each season (mid-January, mid-April, mid-July, and mid-October) over 50 years of the model run from 1959 to 2008 to capture seasonal and interannual variability (not reported here). Higher frequency releases were not considered to be required as spawning times of deep-sea species are generally poorly known. The total number of particles simulated for each case study, for each behaviour, is the number in the final column of Table 1 times 400 (400 = 50 years times 4 seasons per year times 2 launch depths). Polygons for LoVe Observatory (case study 1) and East Mingulay (case study 4) only encompassed a small number of model grid points, 187 and 9, respectively, so additional particles were added to the simulations by creating new particles offset by ±0.3 index units (for both) and ±0.1 index units (only for CS04) to the particles launched exactly at the grid nodes. Rockall Bank (case study 3) and Reykjanes Ridge (case study 9) polygons contained too many points so the numbers of particles were cut down by sub-sampling every 4th node and every 10th node respectively.

From the deep-sea literature^18,19, 41–44^ we have identified 5 key larval vertical movement behaviours, and selected ranges of each within the bounds of observed behaviours:

1. Age of maturity at which full swimming ability, or maximum positive buoyancy is reached. Range [0,10 days].
2. Age of competency at which larvae start heading downwards to find somewhere to settle. Range [4 days, 42 days].
3. The peak upward swim (or buoyancy driven) speed achieved after the age of maturity. Speed increases linearly from age zero until the age of maturity. Range [0.2 mm s^−1^, 1.0 mm s^−1^].
4. Maximum downward speed. This is the speed of descent after the age of competency. Range [−0.2 mm s^−1^, −1.0 mm s^−1^].
5. The target depth below the surface at which larvae stop heading upwards. Range [6 m, 120 m], this range was chosen to investigate the possible effects of near-surface wind-driven Ekman layer flows on particle dispersal.

**Figure 2:**
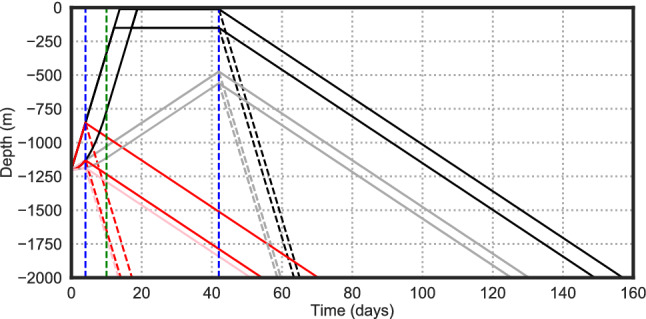
Depth versus time schematic of the 32 larval behaviours modelled (black and red lines). Vertical dashed blue lines are the age of competency. Vertical dashed green line is the later age of maturity (the earlier one is zero). Steep/shallow upward/downward gradients show the fast/slow upward/downward swimming speeds. Near-surface horizontal sections on the black lines show the target depths (many particles may never reach these levels). Many of the 32 line sections overlay each other. Once particles reach the bed they continue close to the bed. Every particle survives for 6 months (185 days).

In a systematic experiment, with each trait varying independently, this gives a total of $2^5 \left( = 32\right)$ tracking experiments each with 9.2 million active particles. The behaviours are shown schematically in Fig. 2, a full list is in Table 2. A further control data set is 9.2 million passive (purely advective) particle tracks.

**Table 1:** Numbers of particles released at each release time, for each behaviour, by Case Study (CS) region.

	Case study	Focus ecosystems (CWC, cold-water coral)	Current and BG sectors*	Bathy min. [m]	Bathy max. [m]	Particles per release
1	LoVe Observatory	CWC reefs, sponges	F, OG, T	20	2000	1683**
2	West of Shetland and West Scotland slope	Sponge grounds	B, F, OG	400	600	259
3	Rockall Bank	CWC reefs, coral gardens, carbonate mounds, sponge grounds, cold seeps	B, F, OG	200	2000	4508***
4	Mingulay Reef Complex	CWC reefs	F, T	100	200	729**
5	Porcupine Seabight	CWC reefs, coral gardens, carbonate mounds, sponge grounds	B, F, OG	200	1200	5295
6	Bay of Biscay	CWC on slope and in canyon settings	B, F	200	2000	1339
7	Gulf of Cádiz, Strait of Gibraltar and Alborán Sea	CWC reefs, coral gardens, sponge grounds	B, F, OG	30	2200	1764
8	Azores	Hydrothermal vents, seamounts, coral gardens, sponge grounds	B, F, M	150	1250	1968
9	Reykjanes Ridge	Hydrothermal vents, CWC reefs, coral gardens, sponge grounds	B, F, M	200	2000	1917***
10	S Davis Strait, Western Greenland and Labrador Sea	CWC reefs, coral gardens, sponge grounds	B, F	200	2000	950
11	Flemish Cap	Coral gardens, sponge grounds	B, F, OG	600	1450	2349
12	US Mid-Atlantic Canyons	CWC reefs on slope and in canyon settings	B, F, M, OG	200	1500	302

*Blue growth sectors: Biotechnology; Fisheries; Mining; Oil and Gas; Tourism.

**Small sites, added extra release points.

***Large sites, reduced number of release points.

**Table 2:**
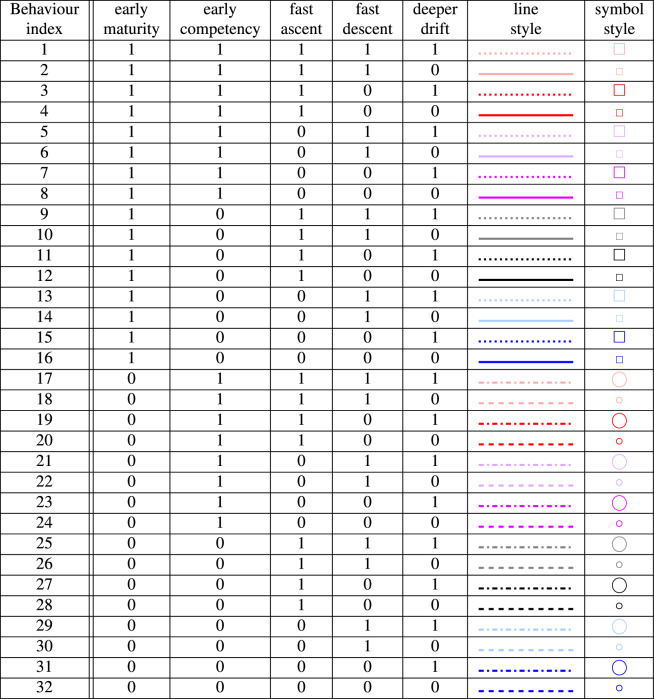
Key to particle behaviours and the line styles and symbols which represent them in Figs. 5, 6. A ‘1’ in the column indicates the behaviour in the header, a ‘0’ indicates its alternative behaviour (here in brackets). Early(late) maturity: 0(10) days. Early(late) competency: 4(42) days. Fast(slow) ascent or descent: $1.0(0.2)\,\hbox {mm\,s}^{-1}$. Deeper(shallower) drift: 120(6) m.

In each run, particles were spawned quarterly over 50 years and each tracked in the model for 6 months (185 days). This duration was chosen based on a summary of published estimates^18^ together with recent estimates of lifespans of over 1 year for some deep-sea species^19,44^. We have not modelled possible larval behaviours dependent on ocean temperature and salinity. We allow particles to cross watermass boundaries freely based on laboratory^44,45^ and field observations^46^ of this behaviour.

### Particle track visualization and postprocessing

Particle tracks are stored in netCDF format and the post-processing and visualization of the larval simulations has been performed in Python using NumPy, Matplotlib, and cf-python packages as well as with the tcdf particle track visualization toolkit^22,47^. All tracks are accumulated and at intervals of 5 days of particle ‘age’ (time since particle release), 2D (x–y) histograms of modelled particle positions are constructed using bins of ${0.25}{^{\circ }}\times {0.25}{^{\circ }}$ horizontal resolution. Two types of histograms are constructed; the first type includes all particle positions in each bin (depth-integrated) and the second type uses only the particle positions within the two bottom grid cells (depth-selective) to highlight where larvae may settle^48^. Area of spread is quantified as the smallest area within the contours enclosing 100%, 95%, 90%, 80% and 50% of the particle positions^16^. Note that the spreading areas reported in the main article and in the area metrics presented below are always based on the 95% confidence contour, the other contours are present in Supplementary Figs. S13–S24.

### Particle track ensemble metrics for quantification

**Figure 3:**
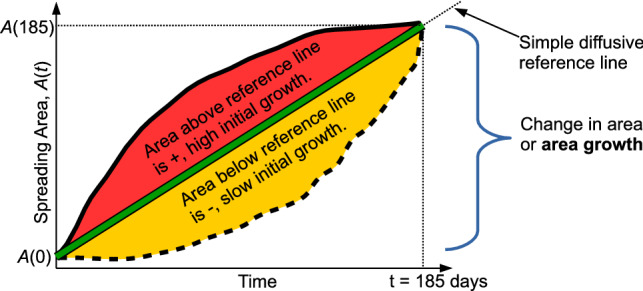
Schematic of the definition of the area growth, *G*, and relative area curvature, *C*, metrics used in quantifying particle track ensemble patterns.

In order to quantify the total spreading and relative change in regime experienced by particles, we formally define two metrics that describe the time evolution of an ensemble of particle tracks (Fig. 3). The first, the area growth, *G*,

1 $$\begin{aligned} G = A(\text {185 days}) - A(\text {0 days}), \end{aligned}$$

is the difference in areas, *A*(*t*), contained within the 95% confidence contour of the depth-integrated particle position probability map after 185 days and at the time of launch. The second metric, the relative area growth curvature, is the normalized area between the curve *A*(*t*) and a straight-line diffusive approximation from $A(\text {0 days})$ to $A(\text {185 days})$, *a*(*t*),

2 $$\begin{aligned} C = \frac{\int _{t=0}^{t=185} A(t) - a(t) dt}{\int _{t=0}^{t=185} a(t) dt} = \frac{\int _{t=0}^{t=185} \epsilon (t) dt}{\frac{1}{2} G \Delta t}, \end{aligned}$$

where $\epsilon (t)$ is the instantaneous difference between the area growth curve and the simple diffusion reference line and $\Delta t$ is 185 days. Positive (or negative) values of *C* mean that the spreading area initially grows faster (slower) than the simple diffusive reference and then slows down (speeds up). Values close to 0 suggest that the area growth is very similar to the simple diffusive reference line with minimal change in flow regime. Since a straight line is the reference within *C* and there is no contraction of area with time, *C* is always between -1 and 1.

Finally, for each launch site, we calculate the along-bathymetry retention after 185 days, *R*, for each behaviour, by dividing the number particles near the bed and between the 200 m and 2200 m isobaths (from the depth-selective histograms), by the total number of particles. This metric does not account for individual species requirements for narrower depth or temperature ranges, the heterogeneous distribution of suitable or unsuitable sea floor substrates, other environmental cues, mortality, or predation, so it is an overestimate of settling and larval survival. However, because bathymetry is the dominant filter, and ocean currents are strongly steered by bathymetry, *R* provides a rough quantification for the degree to which larvae follow the main currents of the Subpolar North Atlantic Ocean.

## Results

### Dispersal rates

**Figure 4:**
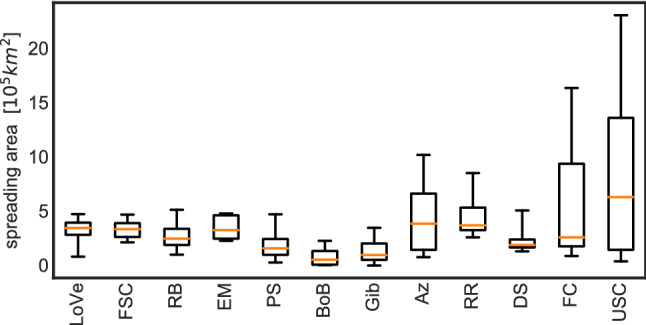
Box plots of the smallest area containing 95% of particles after 185 d over all behaviours for each source region. The statistics presented here are constructed from the values of area growth, *G* (see Methods), determined from each particle tracking run at each launch site. Box plot whiskers show the full range, boxes show the central 50% of results and horizontal orange lines are the median. For source area abbreviation key see Fig. 1 caption. Ratios of maximum:minimum spreading areas are LoVe:6.3, FSC:2.5, RB:5.3, EM:2.1, PS:18.8, BoB:>100, Gib:>100, Az:13.3, RR:3.4, DS:3.8, FC:23.0, USC:72.1.

In the range of values tested in our simulations, larval vertical movement and position profoundly affect dispersal, with most modelled source regions recording associated variations in area of spread of more than one order of magnitude (Fig. 4). The strength of this effect is location-dependent: the influence of particle behaviour on dispersal varies between a doubling of spreading area (East Mingulay), to a factor of over 50 (US Canyons, Bay of Biscay), between the weakest and strongest dispersal. Maximum recorded spreading varies by a factor of 10 between the least dispersive area (Bay of Biscay) and most dispersive (US Canyons) regions, reflecting the local dynamics.

**Figure 5:**
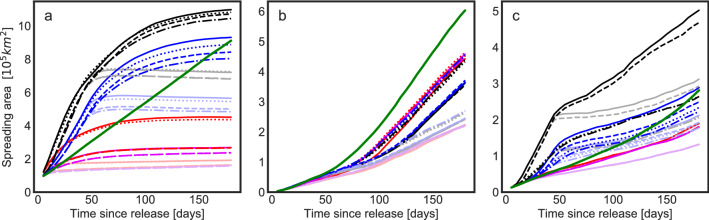
Area occupied by the densest 95% of the released particles versus particle age for (a) Azores (b) East Mingulay and (c) Davis Strait particle releases. Behaviours are keyed by colour and line style. The major behaviour groupings are blacks and blues (reds and purples) $\implies$ late competency (early competency); Dark(Pale) $\implies$ slow descent (rapid descent); Blacks and reds (blues and purples) $\implies$ rapid ascent (slow ascent). Full key in Table 2. Green lines show the near-linear growth of the area of spread of passive larvae which continue to disperse more rapidly at later ages as they do not descend to the bed.

Figure 5 shows the area over which larvae spread for three representative sites; Supplementary Figs. S1–S12 show these areas for all sites. A reference point in each figure is the dispersal of passive particles in our control experiment (green lines in Figs. 5, S1–S12); the area of spread of passive particles increases roughly linearly in time, although with different rates for different release sites. This approximately constant area growth rate for passive particles is consistent with 2-dimensional dispersal by turbulent flow with a constant, spatially uniform dispersal coefficient. While the ocean is far from such an idealized system^49–51^, the slopes of the green lines are a baseline that highlights when the actively swimming larvae disperse faster or slower than passive particles. The time-variable rates of passive particle spreading differing notably from a constant rate (for example increasing passive dispersal rates for East Mingulay, Fig. 5b) is symptomatic of particles moving from one dynamic regime to another, encountering more turbulent regimes or shears in the flow. The quantification of this regime transition is formalized with the relative area growth rate curvature, *C* (see Methods). Note that while for passive particles observed spreading areas increase approximately linearly with time, *t*, spreading radius—perhaps more relevant to connectivity studies on quasi-linear structures like the mid-ocean ridges or continental slope—increases more slowly with $\sqrt{t}$.

### Influence of specific particle behaviours on dispersal

The fastest dispersing modelled larvae exhibit a general pattern; they tend to spread faster than passive particles for about 50 days and then disperse slower than the passive particle control experiment (Fig. 5a, c). This 50 day time scale reflects the 42 day late competency behaviour as larvae maintain their position in the more dispersive upper waters and then actively descend into deeper, more quiescent waters. The East Mingulay site (Fig. 5b) is exceptional compared to other sites primarily because the site is on the shelf, and so all particles at this site are launched shallower than 200 m while other sites spawn larvae in a range of depths from 200 m down to 2200 m. The result is that at East Mingulay there is no clearly faster initial spreading due to upward-swimming particles being in the upper waters. On the other hand, faster upward swim speed can be associated with wide dispersal for the deeper source regions, reflecting the reduction in time to reach the surface. The influence of target depth on dispersal is larger where there is stronger vertical structure in the near-surface currents, for example in the near-shore surface salinity stratification around the Labrador Sea (Davis Strait, Fig. 5c).

**Figure 6:**
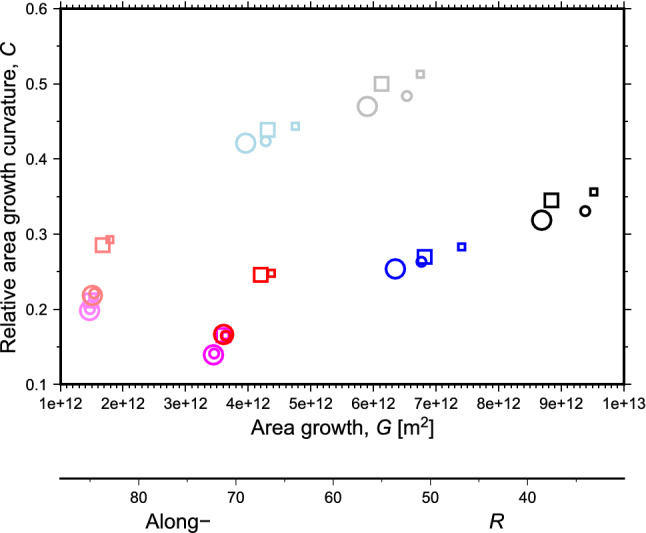
Spreading area metrics for each larval behaviour. Histograms of particle positions from all launch sites were summed and the resulting area growth after 185 days, *G*, is plotted versus the relative area curvature, *C* for each combination of larval behaviours. Symbol definitions are in Table 2. The along-bathymetry percent retention pseudo-x-axis is constructed using the approximately linear relationship between area growth and percent retention (see Supplementary Fig. S27 online). The pseudo-axis gives an approximate representation of whether different behaviours are more likely to result in high proportions of similar-depth settling larvae (at low *G*) or risk losing larvae to unsuitable depths (at high *G*).

While these individual cases are illustrative, the spectrum of impacts of active swimming behaviours on larval spreading can be more systematically quantified by plotting the metrics of area growth, *G*, versus the relative area curvature, *C* (Fig. 6, see Methods for metric definitions). There are four important patterns in Fig. 6, which integrate over all launch sites, that are also mostly present in a site-by-site decomposition of the data (Supplementary Fig. S25). The first is that symbols of different shapes and sizes, but the same colour, tend to group closer together than symbols with different colours. This pattern shows that the larval maturity time (squares versus circles) and target near-surface drifting depth of larvae (big versus small symbols) have smaller impact on larval spreading than the other behaviours. The second pattern is that the lighter hued symbols are generally offset with less *G* but greater *C* than the darker hued symbols. This feature means that larvae with a fast descent strategy (lighter hues) do not spread as far but experience a stronger change in dynamic regime than larvae with a slower descent strategy (darker hues). Thirdly, symbols with cool colours (blue and black) are generally offset with both greater *G* and *C* than symbols with warmer colours (red and purple). This pattern demonstrates that early competency (shorter time near the surface, warm colours) is strongly related to more limited spreading. Finally, faster upward swim speed (black versus blue and red versus purple) is also associated with greater *G* and *C*, increasing spreading, but the effect is not as strong as for the competency behaviour.

In summary, the downward swimming speed has the greatest impact on the change in dynamic regime experienced by the larvae while competency time and upward swimming speed tend to impact the total larval spreading area. The other two behaviours, drifting depth and maturity time have much smaller impacts on larval spreading. Finally, we note that seasonal changes in the patterns described above are also minor. More specifically, when Fig. 6 is redrawn in a seasonal decomposition (see Supplementary Fig. S26 online) the same broad patterns for each season are retained and the scatter of points between seasons is roughly the same order of scatter as with the lowest impact larval behaviours (maturity time and drift depth).

**Figure 7:**
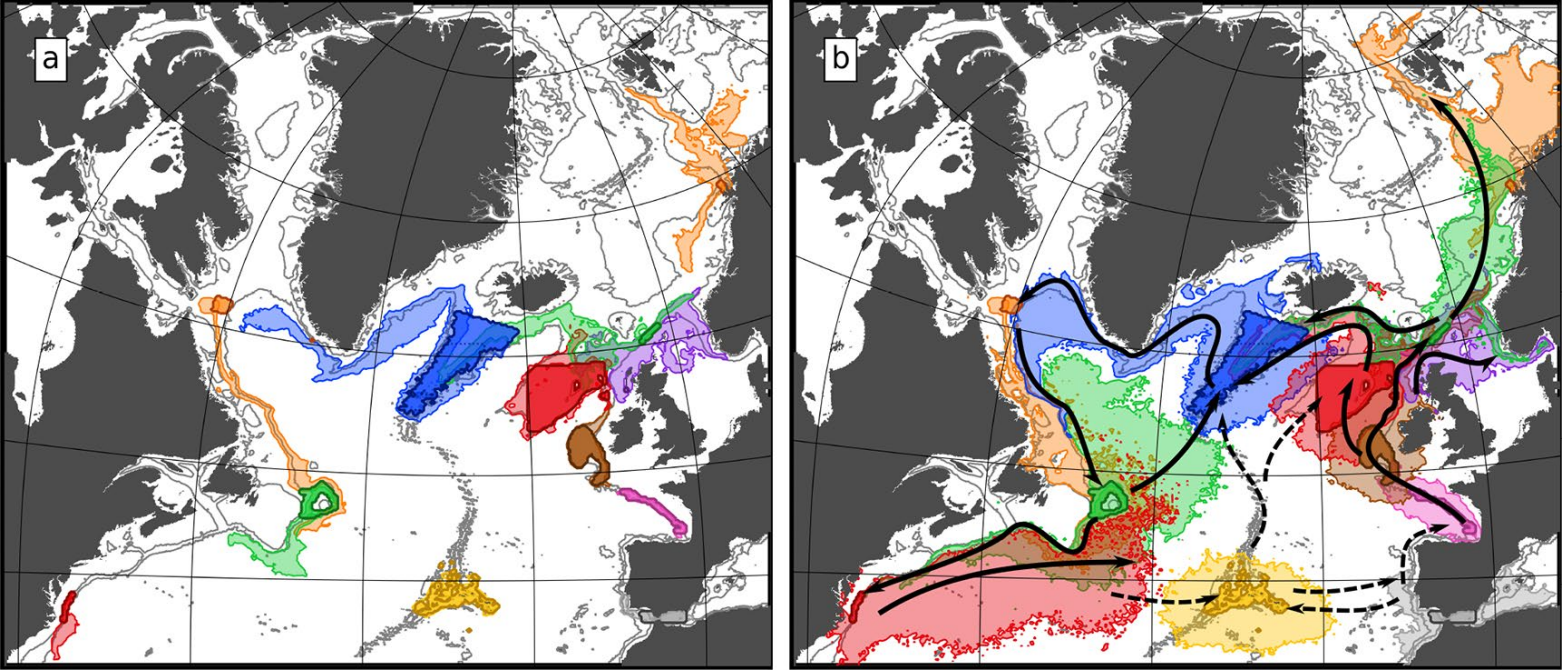
Spreading extent of the densest 95% of modelled particles at an age of 185 days for (a) the generally least dispersive—closest to the bed—particles (case 21 in Table 2) and (b) the generally most dispersive—closest to the surface—particles (case 12 in Table 2). Darker regions show the sources in ATLAS Case Study areas, surrounding lighter regions of matching colour show the extent of spread. Black arrows in (b) show predicted major pathways of connectivity, dashed arrows show more uncertain connectivity pathways reliant on either more dispersive behaviour than modelled or suitable habitat along the path. Background map and depth contours are from the VIKING20 model bathymetry. Figure produced with Jupyter notebook, Python and Matplotlib^22^, arrows added with Inkscape 0.92 (https://inkscape.org/).

### Dispersal pathways

In addition to spreading, particle behaviour also has clear impacts on the dispersal pathways (Figs. 7, S13–S24 online). Here, 2D maps of particle dispersal at age 185 days are presented for two behaviours—the generally least dispersive combination of behaviours that maximizes near-bed ‘demersal’ drifting (behaviour index 21 in Table 2) and the most dispersive combination of behaviours that maximize near-surface drifting (index 12 in Table 2). These two cases approximately bracket the dispersal extremes (little black squares versus light purple big circles in Figs. 6 and S25 online). These maps show that, at the basin scale, following the near-surface strategy results in much wider dispersal than following the demersal strategy.

The interaction of larval behaviour and the vertical variation in flow characteristics around the basin has a clear impact on regional differences in the pathways of larvae with demersal and surface behaviours. Firstly, near-surface drifting allows dispersal into shallower shelf seas which cannot be reached by deeper drifting particles. Secondly, regions with strong vertical structure in the flows result in different dispersal directions for near-surface and demersal drifting particles, this is most evident in the western basin along the USA continental slope and around the Iceland-Scotland ridge. Along the USA continental slope (see also Supplementary Figs. S23, S24 online), demersal drifting particles encounter the southward flowing Deep Western Boundary Current whereas near-surface particles are entrained into the Gulf Stream. Around the Iceland-Scotland ridge, demersal particles are advected westward in the deep overflows whereas near-surface particles flow northwards into the Arctic Mediterranean seas (NE green area in Figs. 7 and S14 online). In contrast, in areas with less vertical variation in flow direction (the Reykjanes Ridge and the Davis Strait) or weaker or variable flows (Bay of Biscay or the Azores), particle pathways are less affected by their behaviour.

Finally, there is a strong, negative linear relationship between the along-bathymetry retention, *R*, and area growth, *G*, (see Supplementary Figs. S27 and S28 online) with greater area growth, *G*, reflecting smaller percentages of along-bathymetry retention, *R*, and vice versa. This relationship is the basis for the *R* pseudo axis on Fig. 6 to illustrate that, broadly across all behaviour combinations, greater spreading area means larvae are more likely to exit the boundary current system and fall outside the ideal depth range for their long-term survival. The weak relationship between along-bathymetry retention, *R*, and relative area curvature, *C*, suggests that how spreading is achieved—either through a short burst near-surface or through longer drifting at deeper levels—has little influence on retention.

## Discussion

Our primary result that, within the range of values tested in our simulations, the vertical position of deep-sea larvae in the water column strongly impacts spreading and pathways, is broadly consistent with previous work. For example, Lagrangian modelling shows that larvae of the deep-sea mussel *Bathymodiolus childressi* drifting in the upper water layers of the Gulf of Mexico could seed most known seep metapopulations on the Atlantic continental margin, whereas larvae drifting demersally cannot^19^. In the northeast Atlantic, *Lophelia pertusa* larvae following vertical swimming behaviour traits predicted from observations in the laboratory^42, 44^ may disperse much more widely than passive larvae^15^. The smallest impact of vertical position on spreading is found for the single on-shelf site studied (East Mingulay), due to its more vertically uniform current regime. Similarly, vertical distribution of drifting larvae has been found to have a secondary role on dispersal in shallow seas^52^.

One of the principal practical motivations for studying dispersal of deep-sea larvae is to assess the connectivity of populations, an important consideration in deep-sea management^2–6^. So, while our main aim is to assess the impact of vertical behaviour on predicted dispersal, it is informative to use our modelled behaviours as an example to highlight, qualitatively, how changes in dispersal might affect connectivity across the North Atlantic basin. We don’t intend this as a guide for planners or policymakers, based as it is on a single PLD.

Our simulation using the most dispersive behaviour and 185 day PLD predicts basin-scale overlap of larval spreading from site-to-site (Fig. 7). This modelled behaviour is based closely on laboratory behaviour estimates for cold-water coral *Lophelia pertusa*^42,44^. The occurrence of seep species of molluscs, crustaceans, and other taxonomic groups across the Atlantic Ocean also suggest such broad connectivity^53^. With this dispersive behaviour we predict connectivity across large gaps spanning abyssal depths, for example releases from the US Mid-Atlantic Canyons show particles almost reaching the Azores, and easily reaching Bermuda, the New England Seamounts and the Corner Rise Seamounts, potential stepping-stone locations. Particles released from the Flemish Cap are predicted to reach potential seamount stepping-stones to the Azores, the Mid-Atlantic Ridge and the southern tip of the Reykjanes Ridge. These modelled connections and multi-generational larval recirculations closely follow the major current patterns in the North Atlantic shown by the mean streamfunction in Fig. 1. With the more dispersive behaviours modelled (Fig. 7b), larvae almost cross eastward from the Azores, and westward from the Gulf of Cádiz, to seamounts on the Madeira-Tore Rise; these links may be a tenuous, bi-directional pathway between the Azores and the European continental slope. Connections from the Azores westward to the North American Atlantic slope look unlikely under all behaviour types tested, but connections from the North American Atlantic slope to the Azores appear possible with more dispersive behaviours and using stepping-stone sites. We do not suggest these long-range connections would be important to population short-term maintenance, but may represent pathways of initial colonisation, or potential recolonisation if populations are lost.

The least dispersive behaviours we model—with early descent to the bed, though still surviving to 185 days (Fig. 7a)—suggest connectivity over only short distances, with no connections across the deep basins. Population connectivity for species with such behaviour would be much more dependent on the small-scale details of habitat distribution. Note that the range of behaviours modelled was designed to investigate the hypothesis that vertical behaviour can affect dispersal, and not to fully represent range of possible behaviours. In particular our assumption of a 185-day PLD is towards the longer end of estimates for deep-sea species^18^.

While we selected behaviour traits from laboratory and field observations, we have not constrained our combined behaviours with the limits of energy stores in lecithotrophic larvae. Neither have we considered higher temperatures near-surface reducing larval life-spans. Both of these biological parameters can be approximately modelled by changing PLD. PLD is often used as single behaviour parameter for dispersal modelling with passive larvae^16^; other results^54^, including those presented here, question whether PLD is a good proxy for more distant dispersal.

Here, we tested 5 other larval behaviours while keeping PLD constant at 185 days. Our last step is to situate our results in the context of PLD. A constant area growth rate (representing simple 2D turbulent dispersal) is used as a reference for computing estimates for the change in dispersive regime experienced by particles (curvature *C*, see Methods). So, by definition, behaviours with small absolute value of *C*, |*C*|, (Figs. 6, and S25 and S26 online) exhibit nearly constant area growth rates, so the area growth *G* will be nearly linearly proportional to PLD. Thus, for behaviours with low |*C*|, the values of *G* reported here, for 185 days, can be scaled accordingly to estimate *G* at PLD other than 185 days with only small errors. By rearranging Equation 2 (see Methods), the average error between the instantaneous *G*(*t*) and $G(185~\text {days})$ linearly scaled by PLD is $\frac{\int \epsilon dt}{\Delta t} = \frac{1}{2}CG$. Furthermore, since the along-bathymetry retention, *R*, shows an approximately linear relationship to *G*, it too can be scaled by PLD. The lower |*C*| behaviours are generally those where particles descend to the bed early or with smaller vertical swimming speeds. For behaviours with larger changes in dispersive regime (larger |*C*|), errors in growth area introduced by curtailing tracks at lower PLD will be larger, making extension of these results to other PLDs inappropriate in these cases.

## Conclusions

We have presented the results from a large-scale, systematic, modelling study investigating how common life history traits of pelagic larvae from deep-sea species affect their dispersal. The major conclusion is that larvae which maximize their residence time near the surface tend to disperse notably more widely than those which remain at depth. In particular, of the 5 behaviours tested here, larval spreading is most sensitive to upward and downward swimming speeds and the time spent near the surface. We also find substantial changes in dispersal pathways reflecting the interaction between larval swimming and vertical changes in currents near the launch sites. This wider dispersal of surface-travelling particles is in addition to that resulting from longer larval lifespan. However, greater dispersal is not always an advantage for larvae; we predict that larger spreading is linked to smaller percentages of the larvae staying within a bathymetry range in which they can survive to adulthood.

The strong relationships reported here between modelled larval spreading, pathways, and active swimming suggest that the lack of data on deep-sea larval behaviour traits^19,41^ is a serious impediment to modelling deep-sea ecosystem connectivity. This limits our ability to develop the well-connected, ecologically coherent marine protected area networks essential to effective deep-sea management and conservation.

The deep-sea regions studied—offshore banks, seamounts and continental slopes at depths of 200 m to 2000 m, rather than abyssal depths—are a current focus of conservation and the search for Blue Growth opportunities while also having unexpected heterogeneity of substrate, habitat^55^ and genetic structure^16^. Our results suggest that the continental slope may provide a continuous arc of connected habitat round the west, north and east of the North Atlantic basin, under many of the behaviour types explored here. Improving our collective understanding of deep sea larval behaviours is a long term project, and with the need for marine management and conservation planning in the short term, it is important to consider a wide range of possible larval behaviours as the context for making marine spatial planning decisions.

## Electronic supplementary material

Below is the link to the electronic supplementary material.

Electronic supplementary material 1

## Data Availability

The histogram data used in the production of the results presented are available from Zenodo, https://doi.org/10.5281/zenodo.3548344^48^. The other large datasets that support the findings of this study are available from the corresponding author upon reasonable request. The VIKING20 model output takes up about 4TB compressed, the raw trajectory output is about 300GB compressed. The modified Ariane particle tracking code, and the scripts and notebooks used to run, postprocess and visualize the trajectory and histogram data, are available at https://doi.org/10.5281/zenodo.3754103^22^.
