# Supplementary material for: Larval behaviour, dispersal and population connectivity in the deep sea
Source: Sci Rep. 2020 Jun 30;10:10675. doi: 10.1038/s41598-020-67503-7 (PMC7326968; doi:10.1038/s41598-020-67503-7)
Supplement: Supplementary file 1 — Electronic supplementary material 1 [file 41598_2020_67503_MOESM1_ESM.pdf]

Supplementary Information for: Larval behaviour, dispersal and population connectivity in the deep sea

Stefan F. Gary   Alan D. Fox *   Arne Biastoch
J. Murray Roberts   Stuart A. Cunningham

April 30, 2020

This PDF file includes:

Figures S1–S28

*Corresponding author: alan.fox@sams.ac.uk

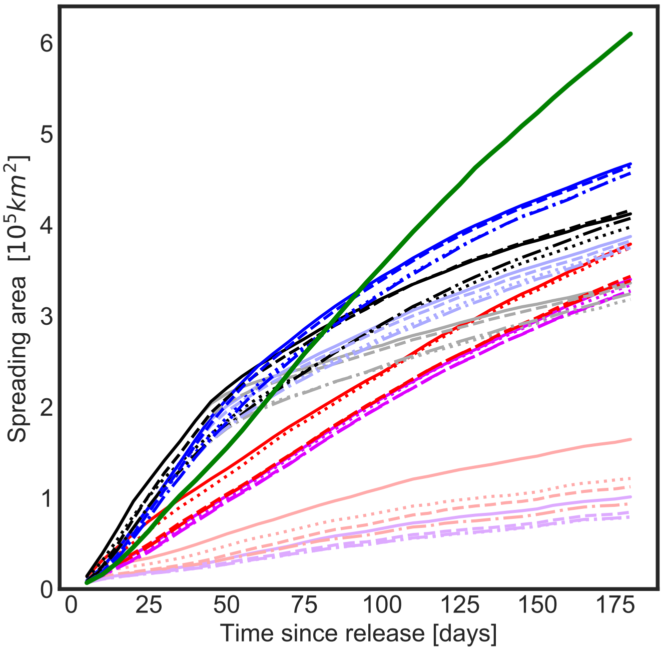

Figure S1: Case Study 01. Minimum area enclosing 95% of particles vs. time. Lines are coloured and styled cumulatively by behaviour (see also Table 1 in main text for key): base solid black, + red – early competence, + blue – slow ascent, + pale – fast descent, + dashes – late maturity, + dots – deeper (120 m target level). The green line represents passive particles, these passive particles do not descend to the bed so more rapid spreading continues throughout.

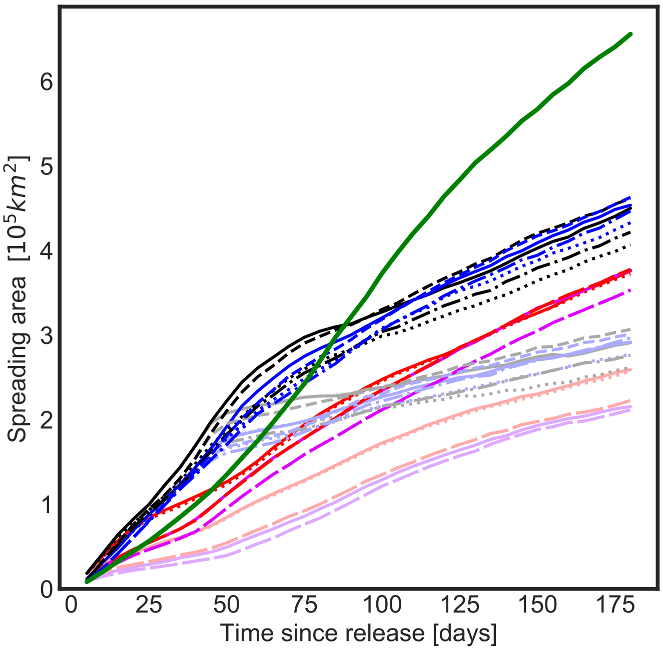

Figure S2: As Figure S1 but for Case Study 02, West Shetland Shelf.

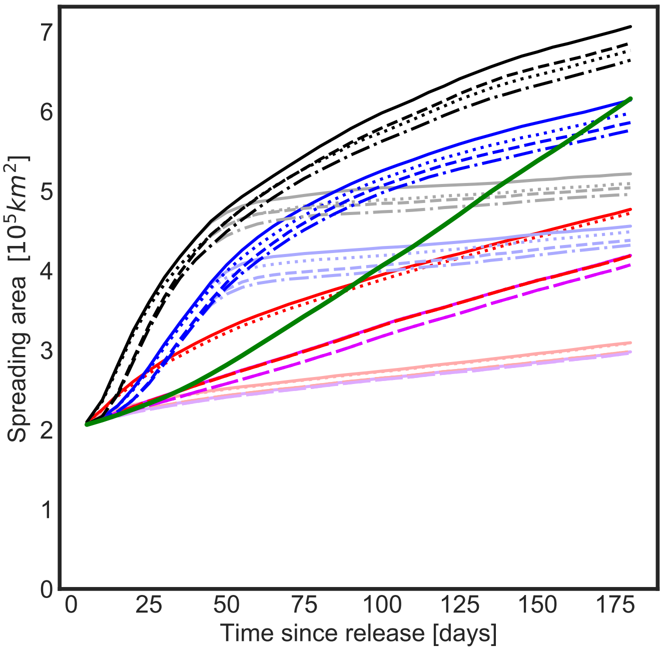

Figure S3: As Figure S1 but for Case Study 03, Rockall Bank.

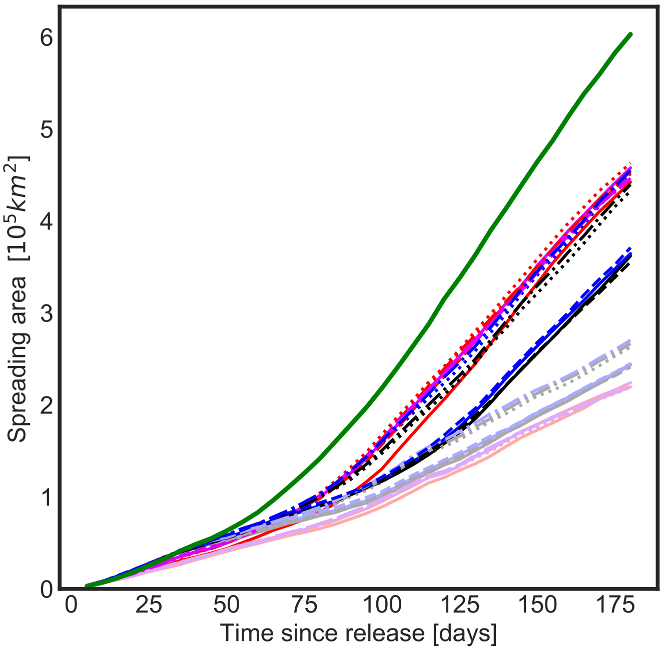

Figure S4: As Figure S1 but for Case Study 04, Mingulay.

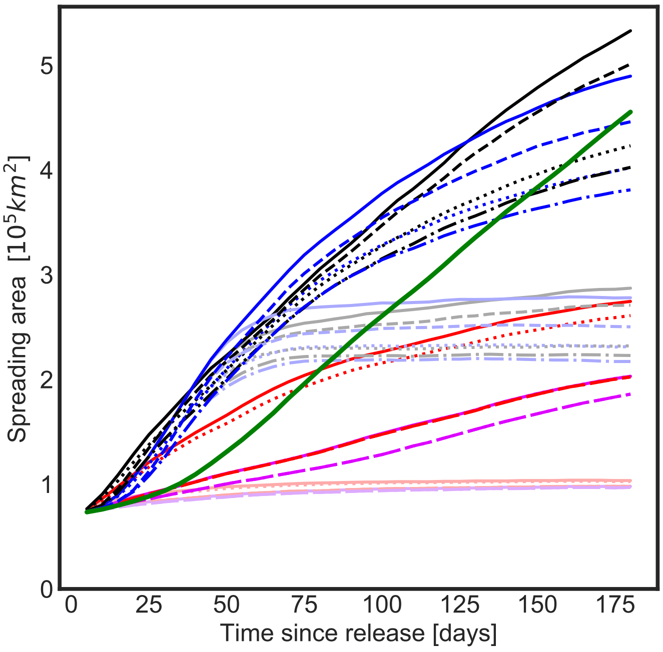

Figure S5: As Figure S1 but for Case Study 05, Porcupine Seabight.

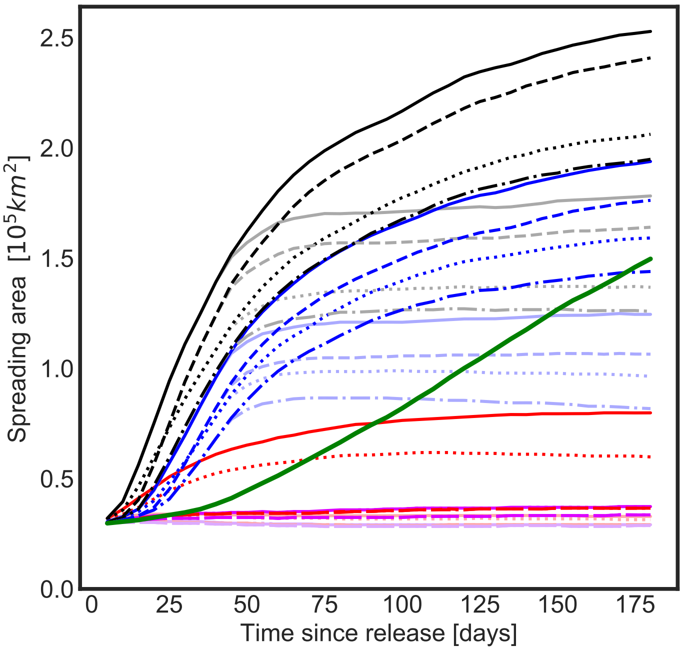

Figure S6: As Figure S1 but for Case Study 06, Bay of Biscay

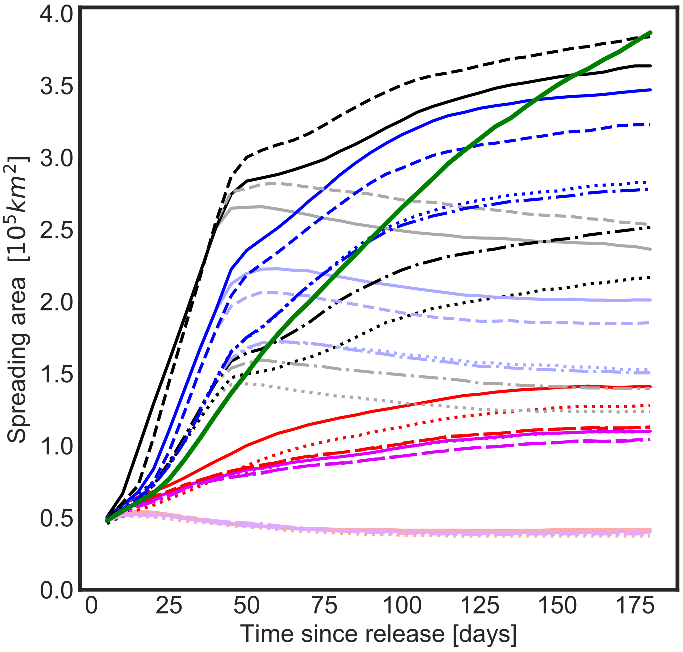

Figure S7: As Figure S1 but for Case Study 07, Gulf of Cádiz and Alboran Sea.

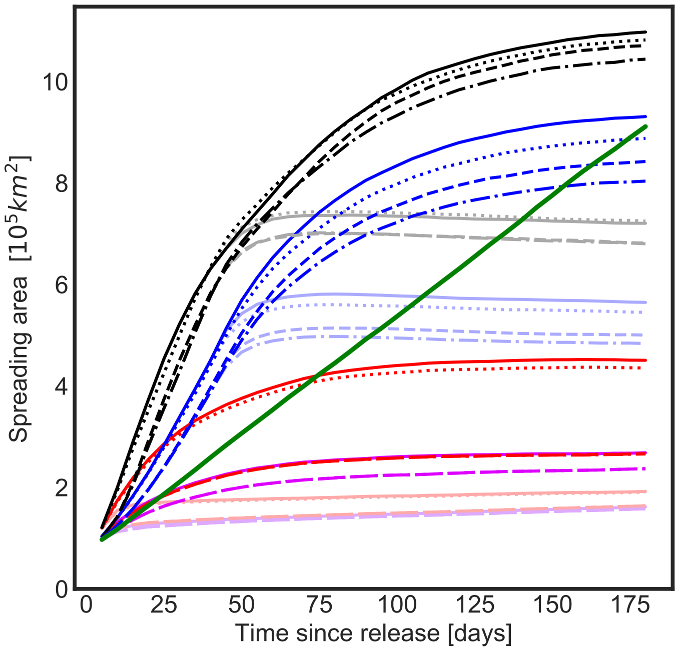

Figure S8: As Figure S1 but for Case Study 08, Azores.

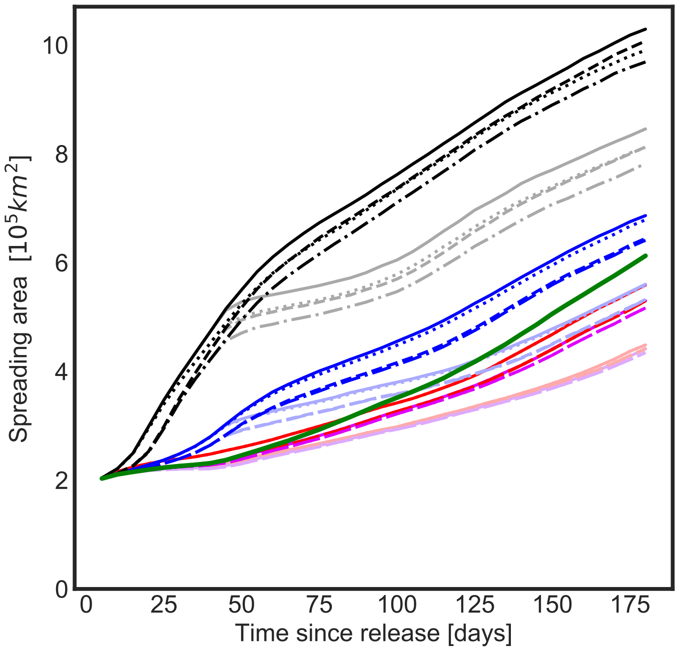

Figure S9: As Figure S1 but for Case Study 09, Reykjanes Ridge.

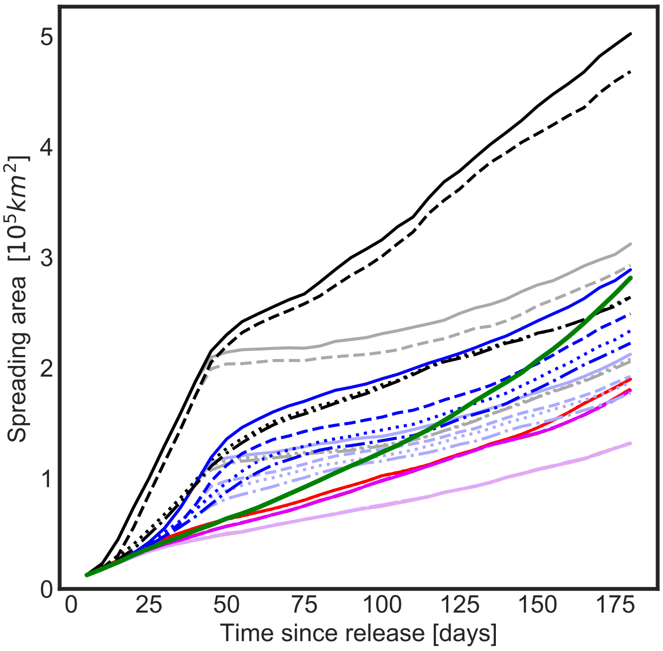

Figure S10: As Figure S1 but for Case Study 10, Davis Strait.

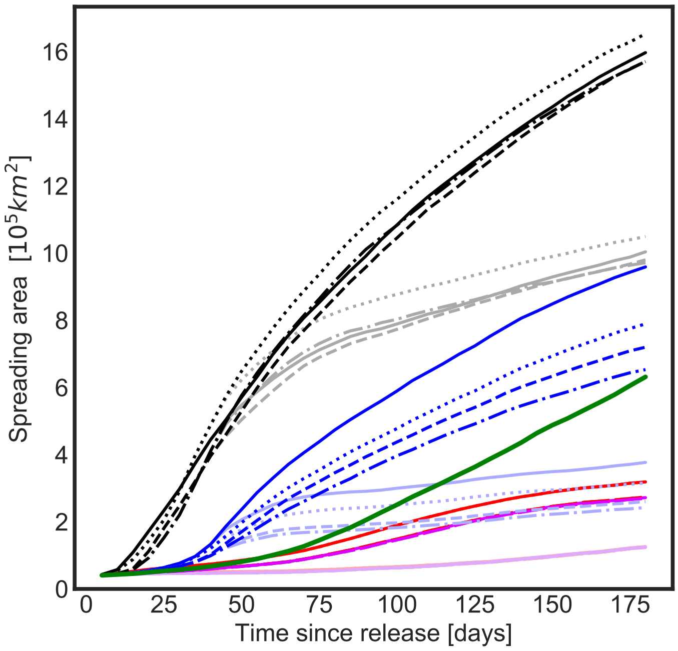

Figure S11: As Figure S1 but for Case Study 11, Flemish Cap

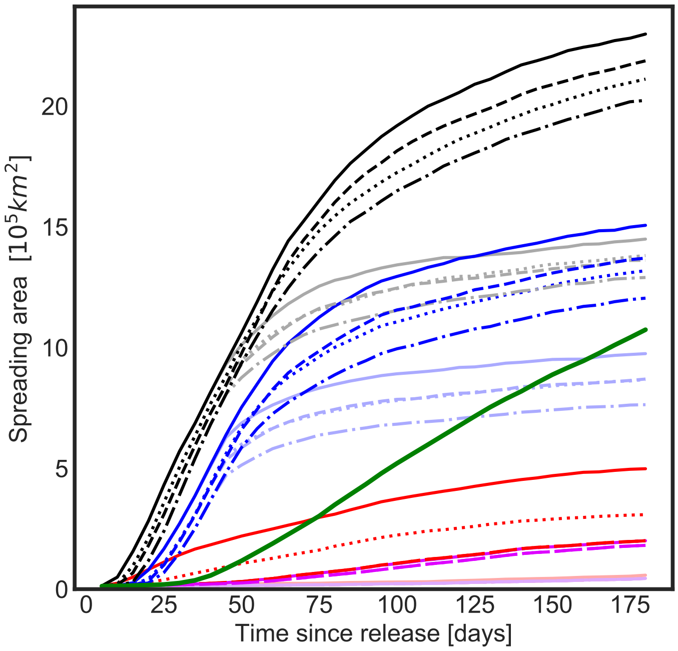

Figure S12: As Figure S1 but for Case Study 12, US Mid-Atlantic Canyons

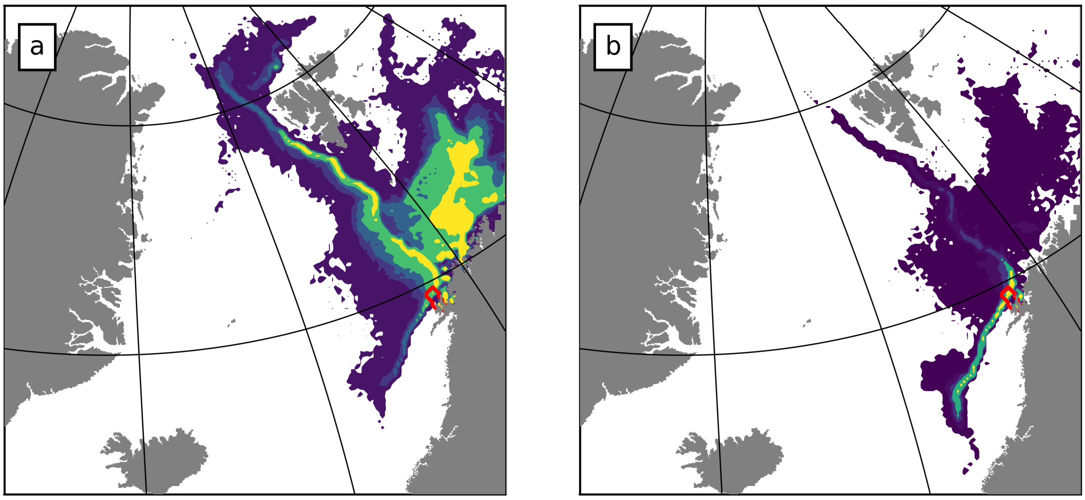

Figure S13: Case Study 01, LoVe: (a) and (b): 2D histograms showing the extent of larval dispersal after 185 days for (a): behaviour type 12 – longest period near-surface, and (b) behaviour type 21 – shortest period above bed. Colours: Yellow – area spanned by the 50% of the tracked particles in the densest part of the distribution, green – 80%, paler to darker blue – 90%, 95%, and 100%. Particles were released from within the red polygon. Background land maps are from the VIKING20 model bathymetry. Figure produced with Jupyter notebook, python and matplotlib, arrows added with Inkscape 0.92 (https://inkscape.org/).

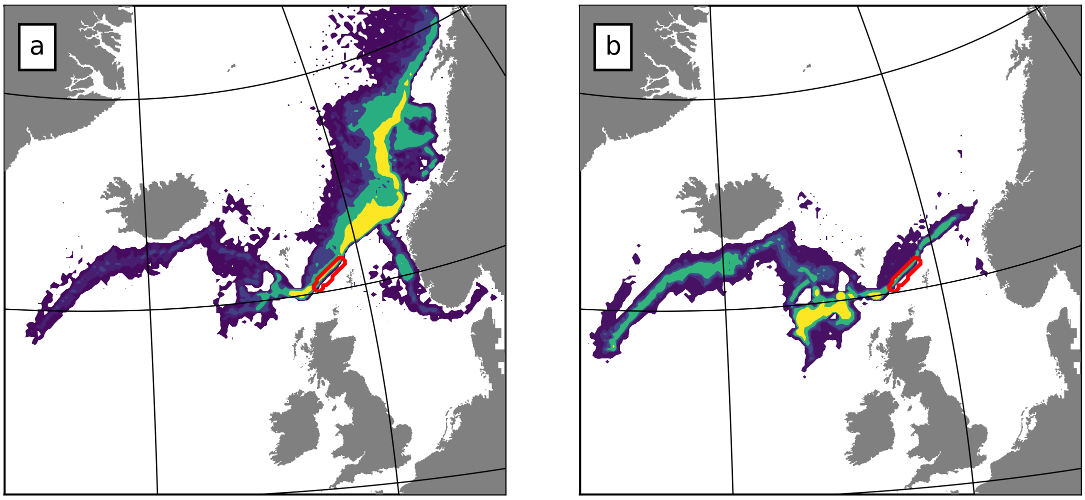

Figure S14: As Figure S13 but for Case Study 02, West Shetland Shelf.

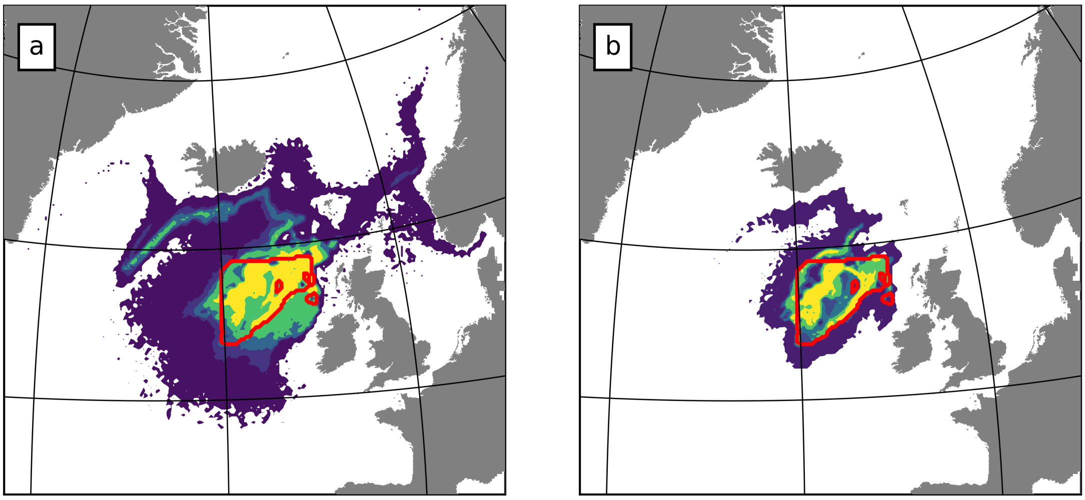

Figure S15: As Figure S13 but for Case Study 03, Rockall Bank.

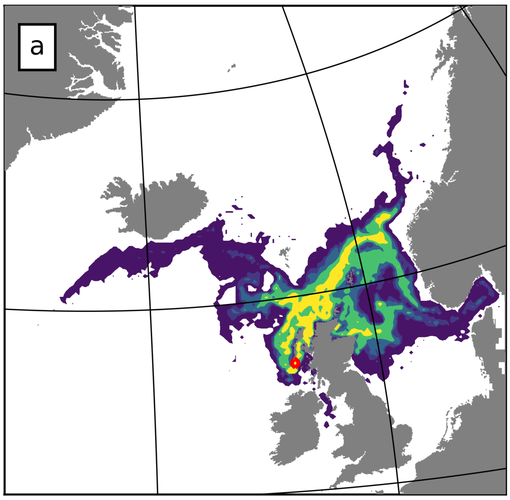

Figure S16: As Figure S13 but for Case Study 04, Mingulay.

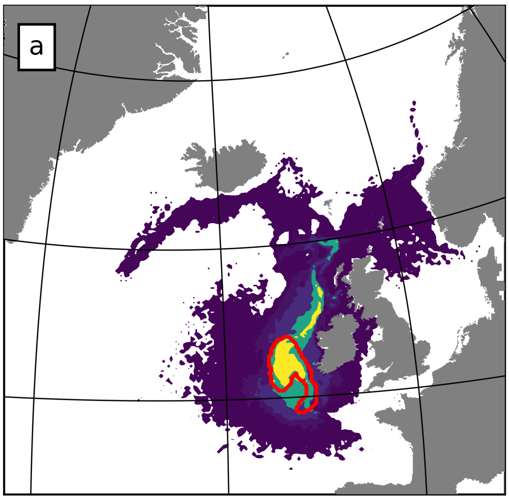

Figure S17: As Figure S13 but for Case Study 05, Porcupine Seabight.

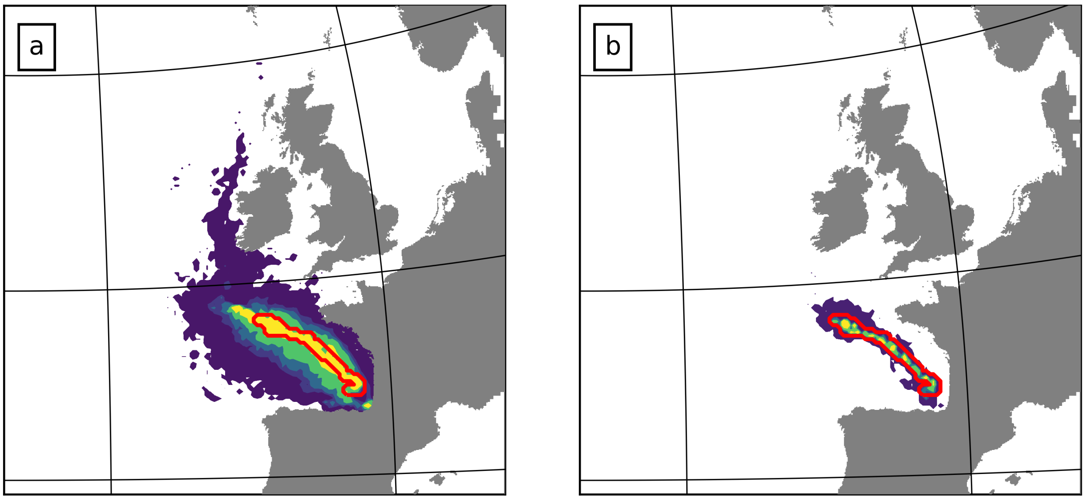

Figure S18: As Figure S13 but for Case Study 06, Bay of Biscay

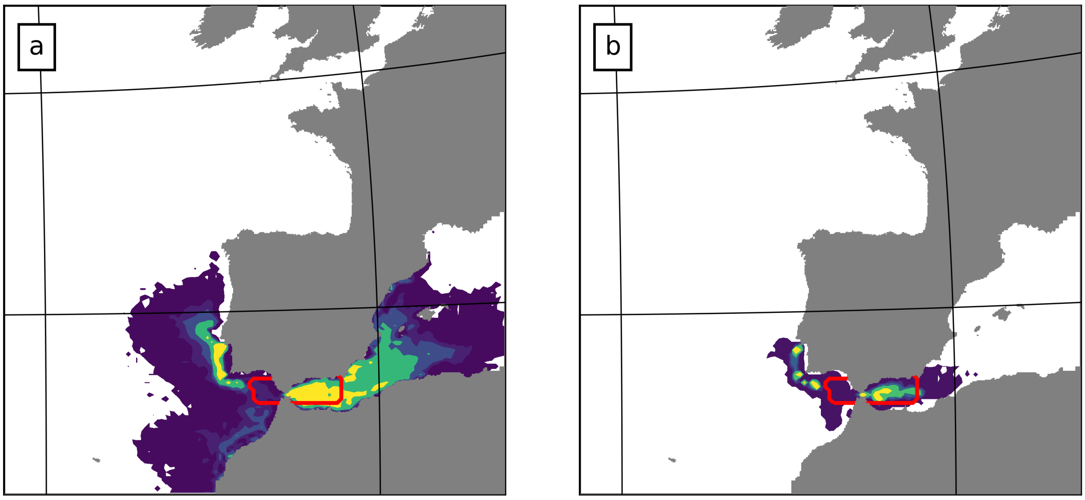

Figure S19: As Figure S13 but for Case Study 07, Gulf of Cádiz and Alboran Sea.

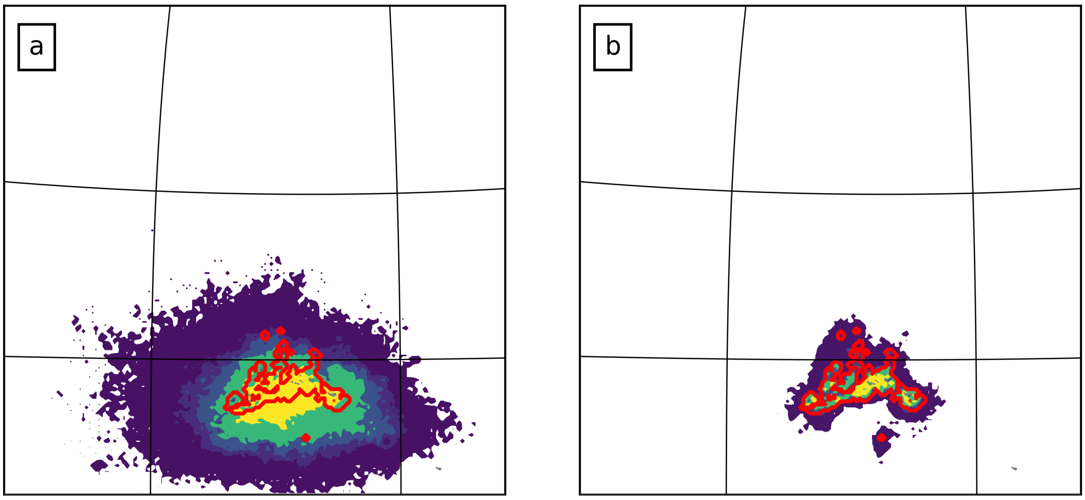

Figure S20: As Figure S13 but for Case Study 08, Azores.

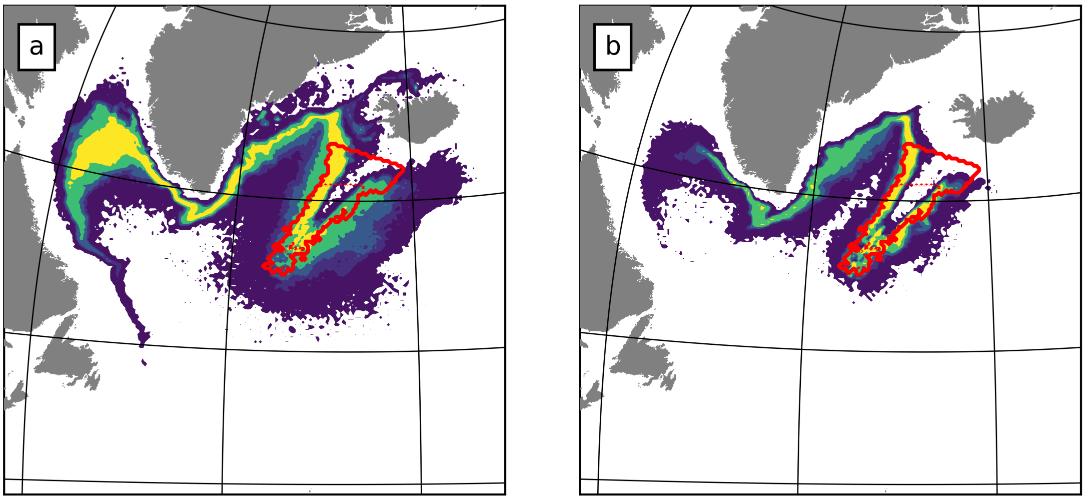

Figure S21: As Figure S13 but for Case Study 09, Reykjanes Ridge.

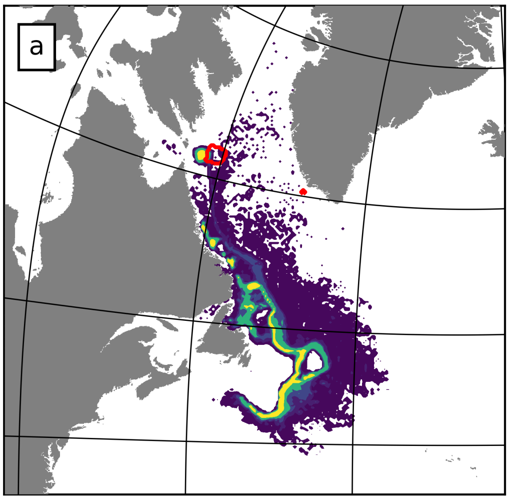

Figure S22: As Figure S13 but for Case Study 10, Davis Strait.

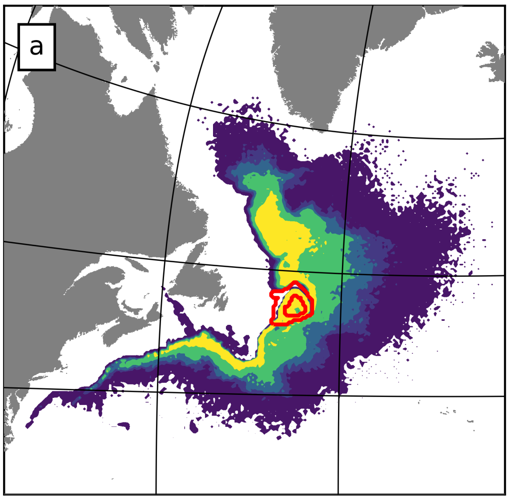

Figure S23: As Figure S13 but for Case Study 11, Flemish Cap

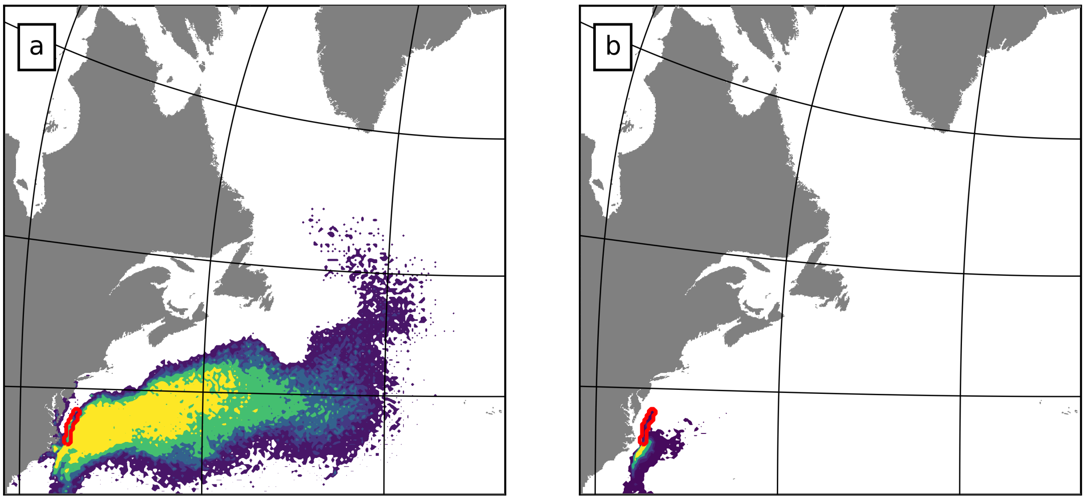

Figure S24: As Figure S13 but for Case Study 12, US Mid-Atlantic Canyons

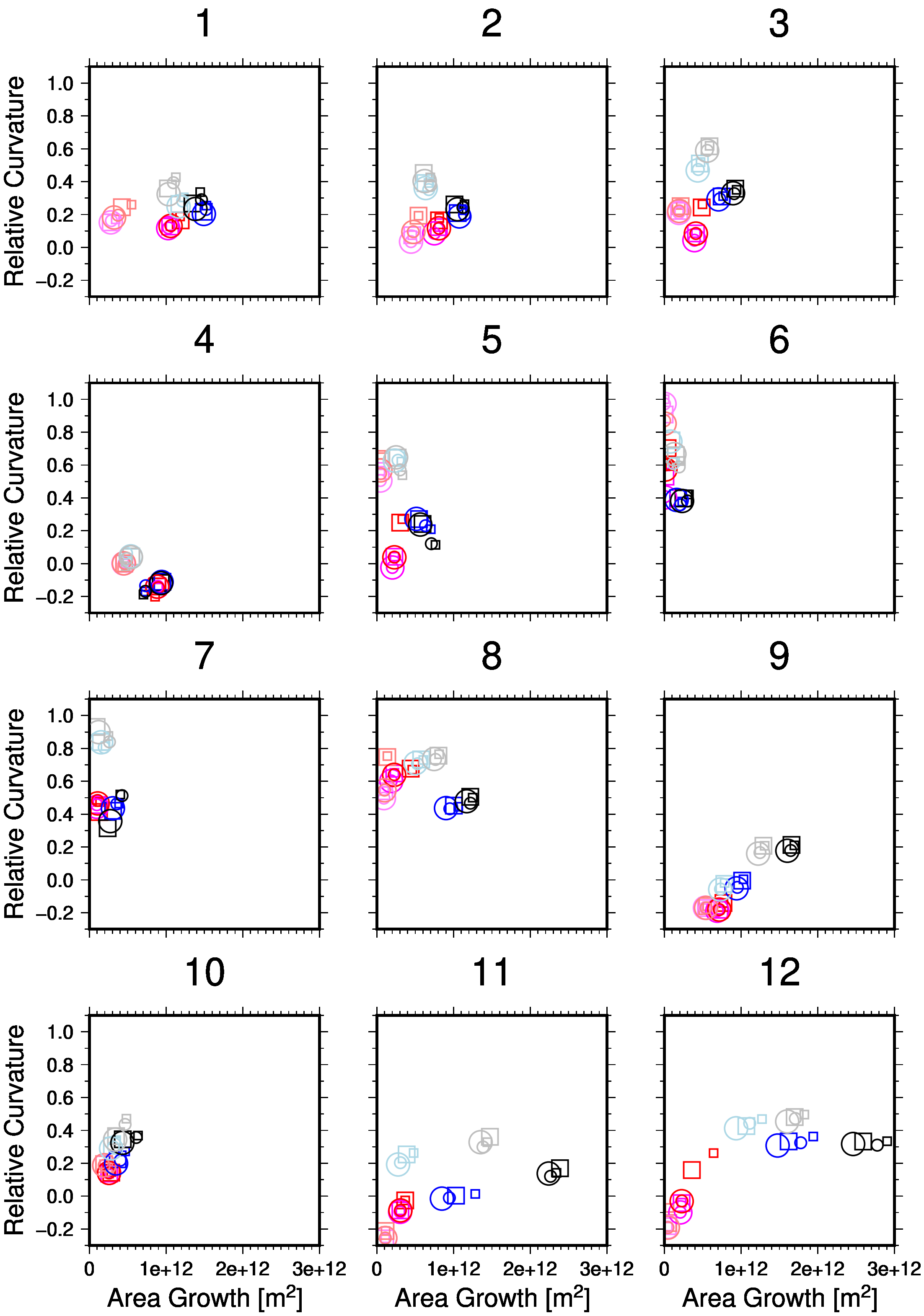

Figure S25: Particle ensemble metrics by site. Same as Figure 6 in main text except metrics are plotted for each of the launch sites. Please see Tables 1 and 2 in main text for the site names and symbol definitions.

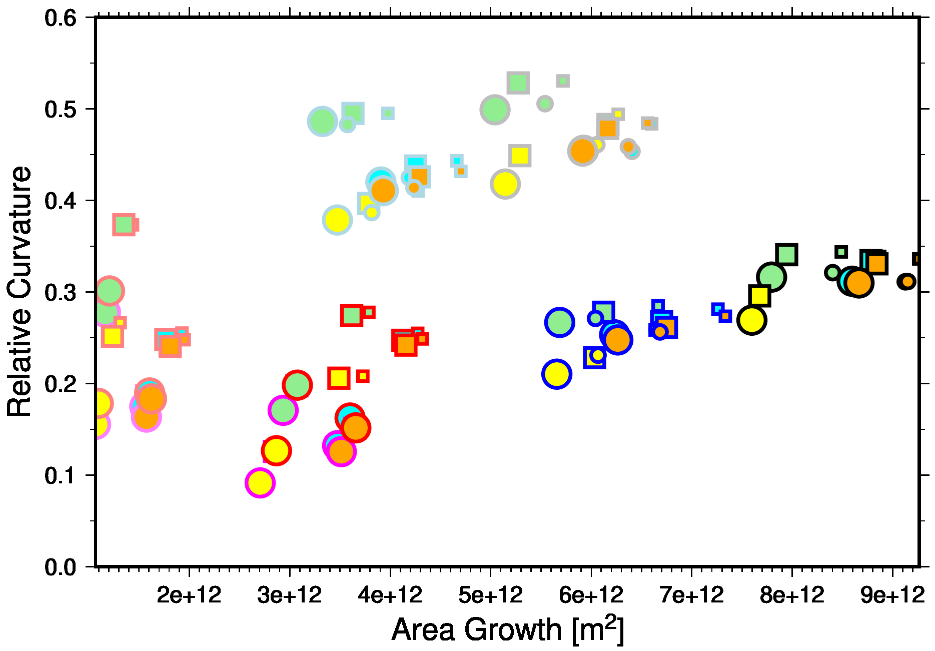

Figure S26: Particle ensemble metrics by seasons. Same as Figure 6 in main text except metrics are plotted for each of the 4 seasons at which particles are launched. The fill colour of each symbol designates the season of launch: winter (cyan), spring (green), summer (yellow), and fall (orange). Please see Table 2 in main text for symbol definitions that set the size, shape, and outline colour.

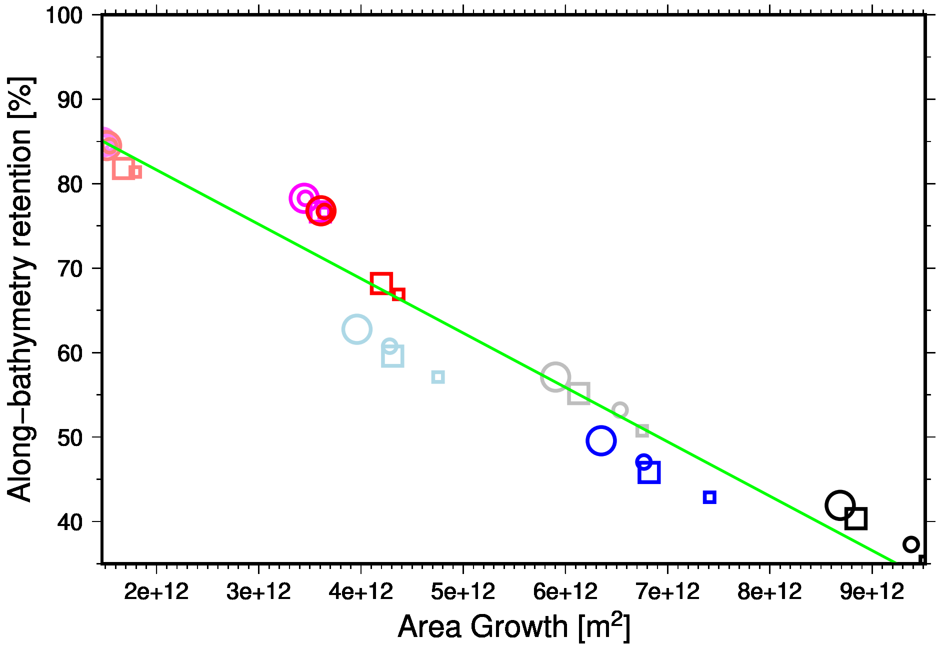

Figure S27: Particle along-bathymetry retention. For all launch sites superposed, the percent of particle positions within two model grid boxes from the bottom of the ocean and between the 200 m and 2200 m isobaths are plotted for each type of larval behaviour versus the same area growth as presented in previous figures. The green line is the linear least squares fit to the plotted data points, $y = mx + b$, where $m = -6.44 \times 10^{-12}\ \mathrm{m}^2/\%$ and $b = 94.5\%$. Please see Table 2 in main text for symbol definitions.

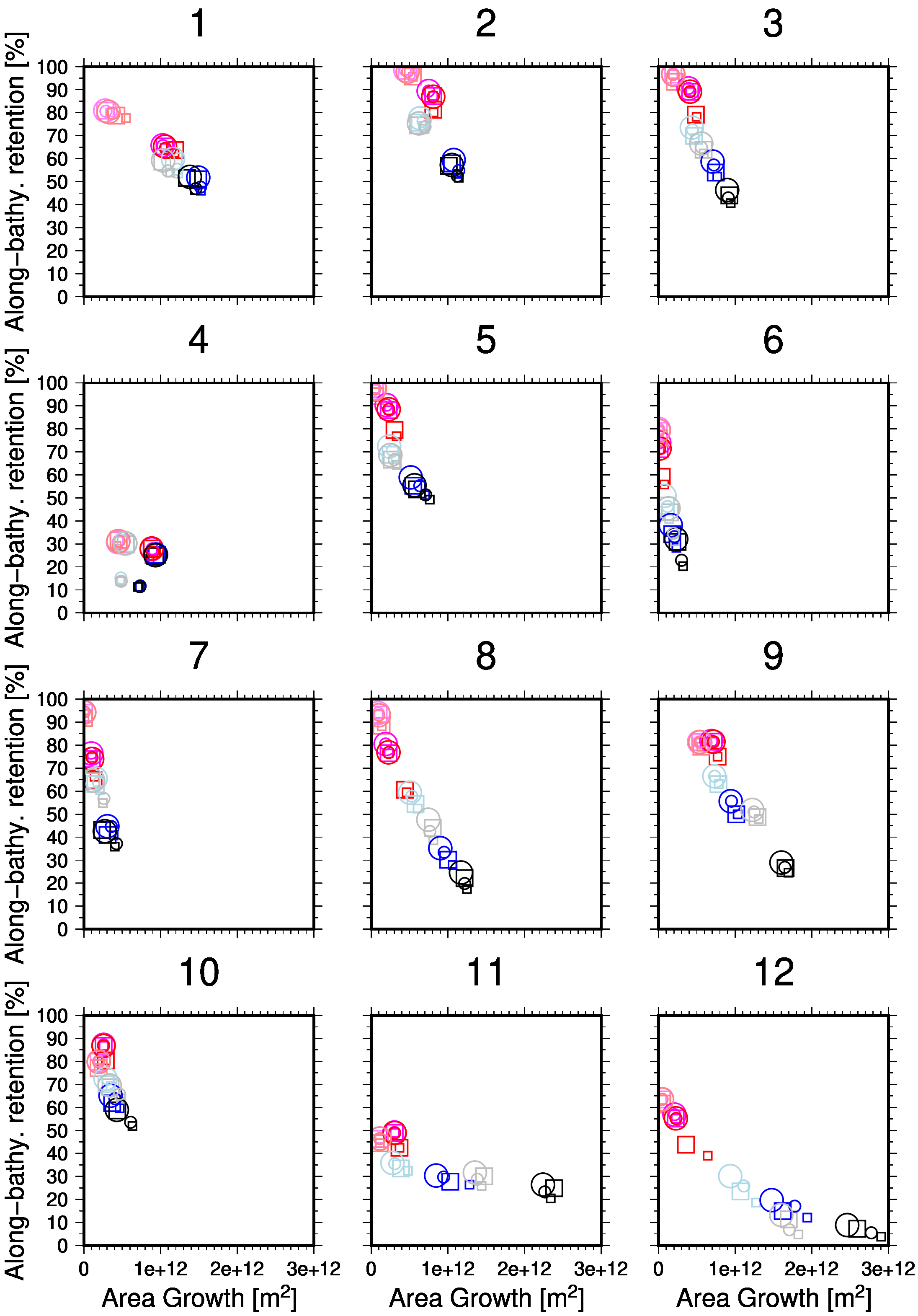

Figure S28: Particle retention by site. Same as Figure S27 except metrics are plotted for each of the launch sites. Please see Tables 1 and 2 in main text for the site names and symbol definitions.
